# Decoding the nucleoid organisation of *Bacillus subtilis *and *Escherichia coli *through gene expression data

**DOI:** 10.1186/1471-2164-6-84

**Published:** 2005-06-06

**Authors:** Anne-Sophie Carpentier, Bruno Torrésani, Alex Grossmann, Alain Hénaut

**Affiliations:** 1 Laboratoire Génome et Informatique, CNRS UMR 8116, Tour Evry2, 523 Place des Terrasses, 91034 Evry Cedex, France; 2 CMI, Université de Provence, 39 rue Joliot-Curie, 13453 Marseille cedex 13, France

## Abstract

**Background:**

Although the organisation of the bacterial chromosome is an area of active research, little is known yet on that subject. The difficulty lies in the fact that the system is dynamic and difficult to observe directly. The advent of massive hybridisation techniques opens the way to further studies of the chromosomal structure because the genes that are co-expressed, as identified by microarray experiments, probably share some spatial relationship. The use of several independent sets of gene expression data should make it possible to obtain an exhaustive view of the genes co-expression and thus a more accurate image of the structure of the chromosome.

**Results:**

For both *Bacillus subtilis *and *Escherichia coli *the co-expression of genes varies as a function of the distance between the genes along the chromosome. The long-range correlations are surprising: the changes in the level of expression of any gene are correlated (positively or negatively) to the changes in the expression level of other genes located at well-defined long-range distances. This property is true for all the genes, regardless of their localisation on the chromosome.

We also found short-range correlations, which suggest that the location of these co-expressed genes corresponds to DNA turns on the nucleoid surface (14–16 genes).

**Conclusion:**

The long-range correlations do not correspond to the domains so far identified in the nucleoid. We explain our results by a model of the nucleoid solenoid structure based on two types of spirals (short and long). The long spirals are uncoiled expressed DNA while the short ones correspond to coiled unexpressed DNA.

## Background

As Lovett and Segall [1] point out in their meeting report on the recently held "Keystone Symposium on Bacterial Chromosomes", we know a lot about the bacterial DNA replication, recombination, repair and other aspects of cell biology, but still rather little about the organisation of bacterial chromosome. The difficulty lies in the fact that the system varies and is difficult to observe directly. A number of different techniques are being employed to answer the problem. The following is meant to give a brief overview and has no claim to be exhaustive:

• **Cytology**-based approaches include the use of DNA fluorescence microscopy, optical sectioning and FISH (fluorescence *in situ *hybridisation). These techniques were applied in order to localise within the cell a set of chromosomal segments [2] or to see the relationship between the shapes of the nucleoid and the underlying arrangements of DNA [3].

• Cunha et al [4] approach the question from a **cytometric **point of view, in order to study the compaction and the internal dynamics of the nucleoid.

• **An example of a classical genetic approach **is the work by Valens et al [5] who have used a site-specific recombination system in order to reveal spatial proximities of distant DNA sites.

• Various **genomic approaches **have been adopted. Some authors, like Audit and Ouzounis [6], have taken a sequence-based point of view, in which they face the issue of gene localisation and orientation using 89 complete microbial chromosomes from eubacteria and archeabacteria. This approach leaves aside any physiology-based consideration.

• Other authors have examined the **physiological constraints **operating placed upon the cell in order to infer chromosomal structure. The idea is that genes which use the same type of resource (e.g. a particular tRNA pool) or which are involved in a part of metabolism that needs a particular environment (e.g. genes involved in sulphur metabolism which is highly sensitive to free radicals) should be in close proximity in the cell, even if they are far away on the chromosome [7,8].

The approaches mentioned above can be spilt in two groups: (i) large-scale analyses, aiming at deciphering the global chromosome organisation; (ii) small-scale analyses, which take a particular point of view (some genes or markers are chosen). The introduction of **microarrays **has added yet another way to study the chromosomal structure, allowing simultaneously the analysis on small and large scales [9]. Microarrays allow the measure of relative expression levels of the whole genome and therefore the identification of those genes that are co-expressed. Usually the co-expressions observations are used to elucidate the structure of operons and other regulatory structures, see for example [10,11].

The present work aims at understanding the nucleoid structure with the help of microarray data. As transcriptionally active DNA is located near the nucleoid surface or on DNA loops extending from the nucleoid [12], the co-expressed genes which are identified with microarrays probably share some spatial relationship.

However, microarrays give significant information only for those genes the level of expression of which varies across experiments. Consequently, the experimental conditions should be diversified in order to obtain a list of gene correlations as exhaustive as possible and thus an accurate image of the chromosomal structure. To this end, we gathered a number of currently available microarray data from the literature. The data were then pooled together, and treated as just one large data set. This "pooling of information" has already been carried out successfully from human expression data for a study of gene function [13], and from yeast or bacterial data for regulation studies [11,14].

We applied this method to two distant bacteria: *Escherichia coli *and *Bacillus subtilis*. Audit and Ouzounis [6] had the same approach, expecting that if observations made on one organism also hold true for the other, it would be reasonable to assume that the inferred chromosomal organisation is indeed a general characteristic of bacteria with double stranded, circular DNA.

## Results

**Figure 1:**
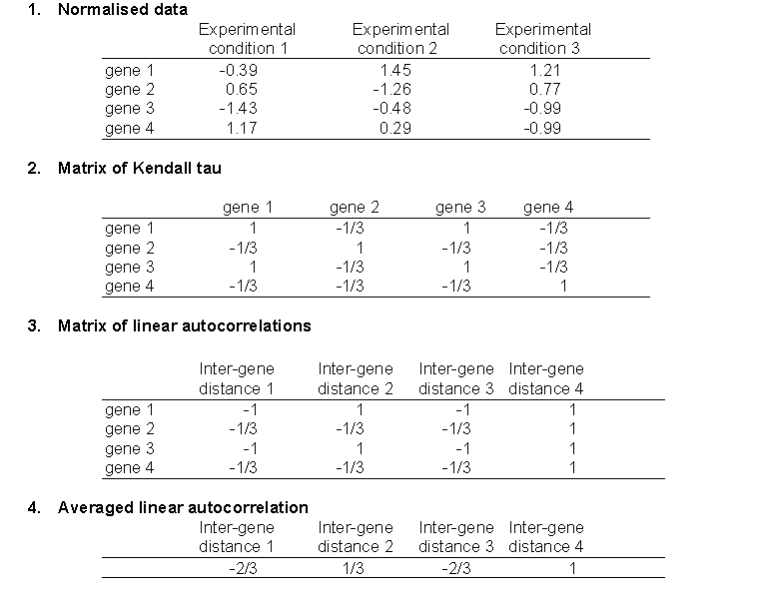
**Illustration of the methodology used in this study. **Example of the results obtained on a hypothetical bacterial circular chromosome model of 4 genes. The gene expression intensities are measured in three experimental conditions. Part 1 is normalised data (mean equal to 0 variance equal to 1) according to experimental conditions. Part 2 is the matrix of Kendall tau (see methods). Part 3 is the autocorrelation matrix with inter-gene distances. Part 4 is the averaged linear autocorrelation.

The aim of this work is to delineate how the co-expression intensities (correlations) of pairs of genes vary as a function of the inter-gene distance along the chromosome. The co-expression intensity for each couple of genes was evaluated with a non-parametric correlation: the Kendall tau [15,16] (see methods and figure 1 part 2) which depends only on the sign of the observed variation and not on its magnitude. Is is thus a "weaker" describer of the data than the linear correlation coefficient (also called Pearson coefficient of correlation) or the Spearman rank correlation coefficient. The Kendall tau points specifically to monotonic correlations. A high Kendall tau between two genes indicates that their levels of expression vary in the same way: when the expression level of the first gene increases, the expression level of the other one increases also.

Then the variation of the Kendall tau coefficient as a function of the distance between genes was measured with a standard linear autocorrelation function [15,16] (see methods and figure 1 part 3). The linear autocorrelation enables to point to regularities in a gene Kendall tau vector and therfore to regularities of expression correlated with particular inter-gene distances.

### *Bacillus subtilis *regularities of co-expression across the genome

**Figure 2:**
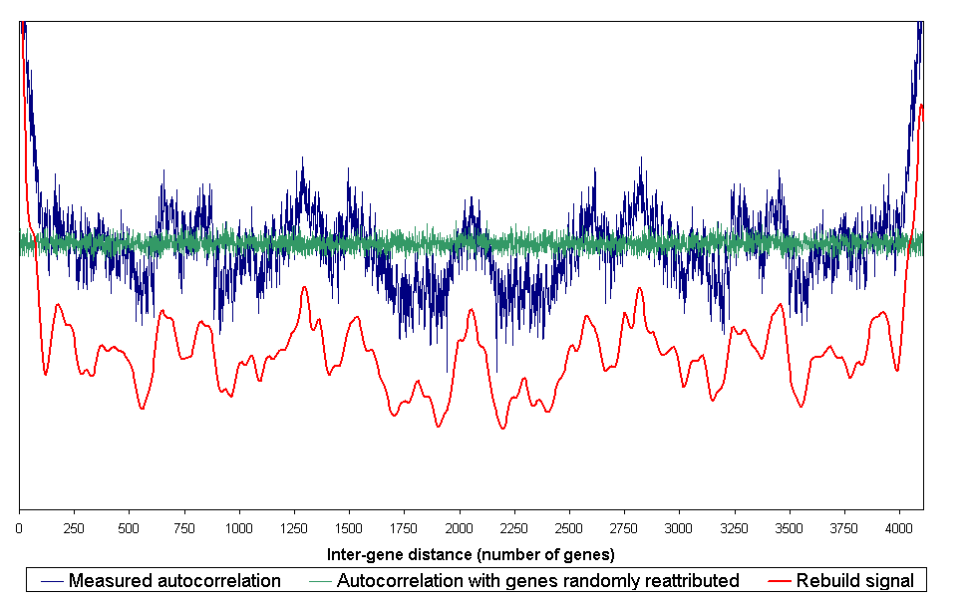
**Long-range averaged autocorrelations in *B. subtilis***. To identify the regularities which are common to most of the genome, regardless of the genes localisation, the autocorrelation vectors of all the genes were summed (blue curve). This global signal shows the averaged autocorrelation regularities as a function of inter-gene distance. The green curve shows the averaged autocorrelation when the genes positions on the genome were randomly assigned. The red curve represents the resultant of four oscillations of periods 600 ± 55, 240 ± 21, 113 ± 21 and 60 ± 6 genes, which were estimated from the averaged autocorrelation deconvolution. The horizontal scale represents the distance between two genes (the difference of their ranks on the chromosome). The green, blue and red curves have the same vertical scale. The red curve was shifted for readability. Whereas the green signal shows no regularity, long-range correlations can be seen in the blue signal (maxima at *ca*. 200, 650, 850, 1300, 1500 and 2050 inter gene distance and minima at *ca*. 550, 900 and 1750–1950).

The analysis of the *B. subtilis *transcription data was performed on a set of 262 experimental conditions gathered from eleven independent experiments measuring expression data over the whole genome. A global view of the regularities of co-expression was obtained by summing up the autocorrelation vectors of all the genes (see figure 1 part 4 and results in figure 2 -blue curve).

The averaged linear autocorrelation of changes in gene expression varies as a function of the inter-gene distance. The green curve in figure 2 corresponds to the averaged autocorrelation evaluated after random permutation of the gene positions on the chromosome. Here the variations are small and independent of the inter-gene distances. Those points where the autocorrelation (blue curve) departs from the random signal (green curve) correspond to couples of genes, for which changes in expression levels are statistically correlated (when the blue curve is above the green one) or anti-correlated (when the blue curve is below the green one).

The autocorrelation function shows regular oscillations at large scale, with maxima at a distance of 200, 650, 850, 1300, 1500 and 2050 genes and minima at a distance of 550, 900 and from 1750 to 1950 genes. Note that the inter-gene distance 2050 corresponds to diametrically opposite genes on the *B. subtilis *chromosome. The autocorrelation function can be seen as the resultant of four oscillations of periods 600 ± 55, 240 ± 21, 113 ± 21 and 60 ± 6 genes. This representation explains 85% of the autocorrelation oscillations (figure 2 – red curve).

**Figure 3:**
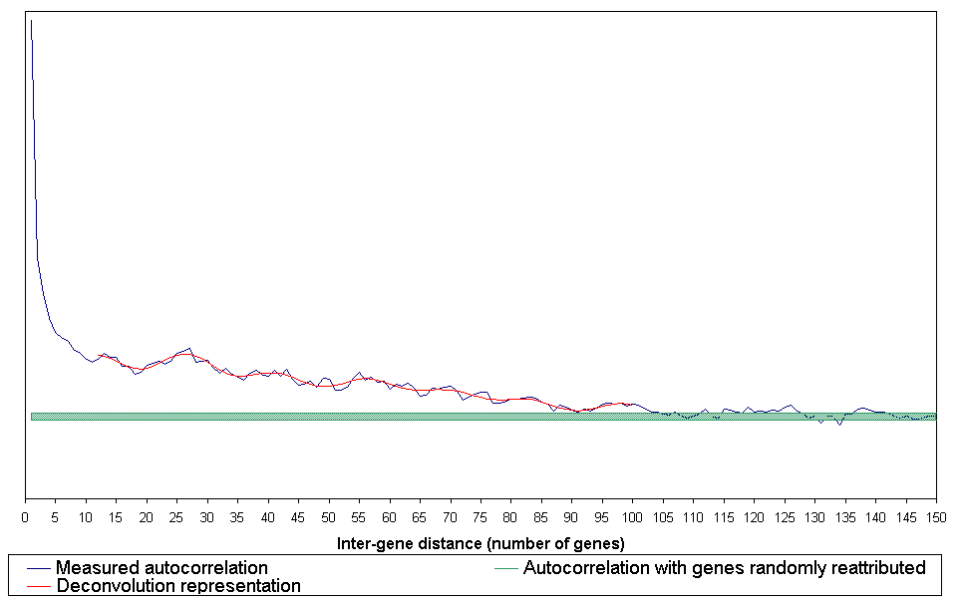
**Short-range co-expression regularities in *B. subtilis***. To identify the regularities which are common to most of the genome, regardless of the genes localisation, the autocorrelation vectors of all the genes were summed (blue curve). This global signal shows the averaged autocorrelation regularities as a function of inter-gene distance. The green zone shows the averaged autocorrelation when the genes positions on the genome were randomly assigned (mean of the random signal ± the root mean square deviation). The horizontal scale represents the distance between two genes (the difference of their ranks on the chromosome). Neighbouring genes on the chromosome show highly correlated variations of expression levels. The averaged autocorrelation of two contiguous genes is 0.4. The signal can be decomposed into two parts: (i) inter-gene distances between 1 and 5–6 genes are characterised by a high autocorrelation, which drops steeply; (ii) beyond 6 genes the autocorrelation shows a regular and slower decrease. The autocorrelation merges with the background noise at an inter-gene distance of about 100 genes (similar to 100 kb). The autocorrelation decrease may be seen as the resultant of a linear decrease and 14.5 ± 1 genes period oscillations (red curve).

The averaged autocorrelation was analysed on a smaller scale with an inter-gene distance comprised between 1 and 150 genes (figure 3 – blue curve). Closely spaced genes on the chromosome show changes in expression levels that are highly correlated. The averaged autocorrelation of two contiguous genes is 0.4. The low-scale autocorrelation can be decomposed into two regimes: (i) inter-gene distances between 1 and 5 (or 6) genes are characterised by a high and rapidly decaying autocorrelation; (ii) beyond a 6 inter-gene distance the autocorrelation shows a regular and slower decay with periodic oscillations of 14 to 15 genes (figure 3 – red curve). The autocorrelation merges with the noise background around an inter-gene distance of 100 genes (corresponding roughly to 100 kb).

**Figure 4:**
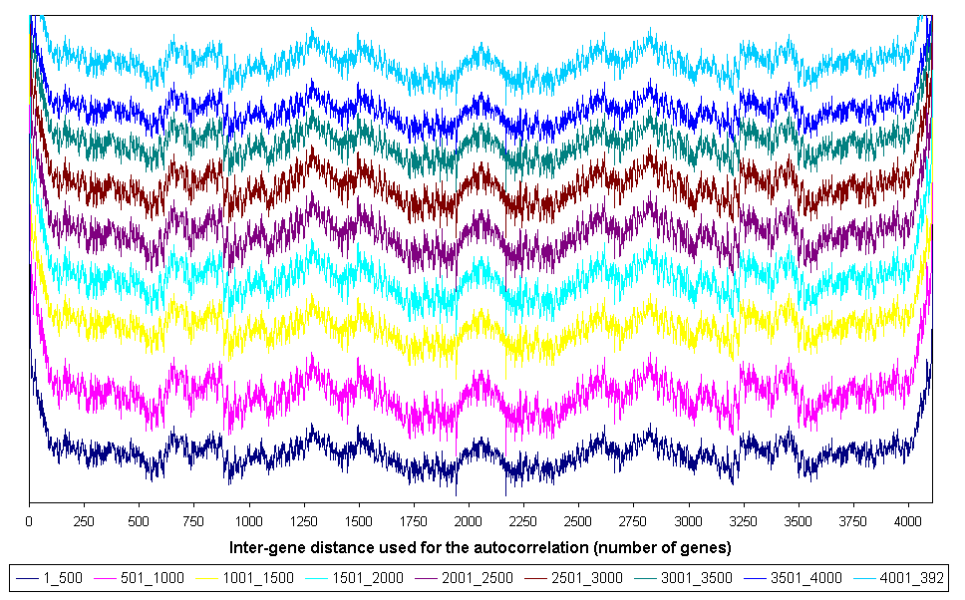
**Partial sums of the autocorrelations in *B. subtilis***. To analyse if the discovered regularities depend on gene position, the autocorrelation vectors of groups of 500 genes were summed up (9 coloured curves). The horizontal scale represents the distance between two genes (the difference of their ranks on the chromosome). All the curves were vertically shifted for readability. The signals show the co-expression regularities according to inter-gene distance. Long-range periodicities are shared by all the signals regardless of the gene groups.

The oscillations of the averaged autocorrelations of the 4108 *B. subtilis *genes shown in figure 2 may result (i) either from regularities specific to some genes or some regions; (ii) or from an overall property that would be shared by all the genes regardless of their positions on the chromosome. In order to ascertain which hypothesis is the correct one, the sums of the autocorrelations of continuous groups of 10, 100 and 500 genes were calculated. All the curves obtained are highly similar (data shown for groups of 500 genes, figure 4). The peaks obtained with these groups of genes are identical to those found in the global signal. Hence they do not depend on any particular position on the genome: in other words, the results show that any gene A has its changes in expression level correlated with the changes in expression levels of those genes that are 200, 650, 850, 1300, 1500 and 2050 genes apart and anti-correlated with those that are 550, 900 and 1750–1950 genes apart. This property is independent of the position of gene A.

### *Escherichia coli *regularities of co-expression across the genome

The same work was performed on *E. coli *with a data set of 106 experimental conditions. This data set is therefore smaller than that used for *B. subtilis*. In addition there are more missing data for *E. coli *than for *B. subtilis*.

**Figure 5:**
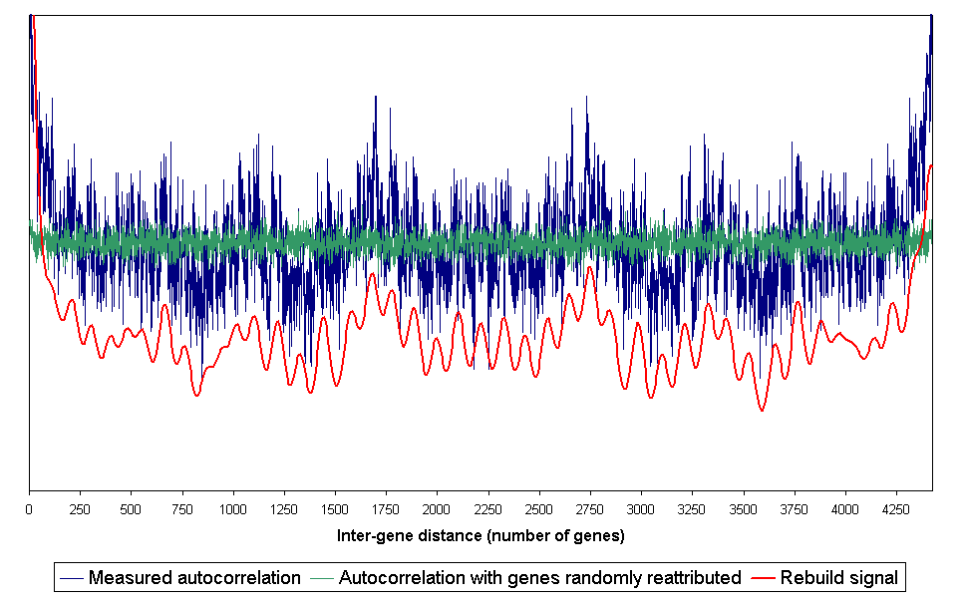
**Long-range averaged autocorrelation in *E. coli***. To identify the regularities which are common to most of the genome, regardless of the genes localisation, the autocorrelation vectors of all the genes were summed (blue curve). This global signal shows the averaged autocorrelation regularities as a function of inter-gene distance. The green curve shows the averaged autocorrelation when the genes positions on the genome were randomly assigned. The red curve represents the resultant of two oscillations of periods 557 ± 30 and 100 ± 18 genes, which were estimated from the averaged autocorrelation deconvolution. The horizontal scale represents the distance between two genes (the difference of their ranks on the chromosome). The green and blue curves have the same vertical scale. The red one is on a scale, which is moved down for readability. Whereas the green signal shows no regularity, long-range periodicities can be seen in the blue signal (maxima at *ca*. 200, 650, 1100, 1400 and 1700 and minima at *ca*. 850, 1380 and 2180).

Figure 5 represents the variations of the averaged autocorrelation of all the genes as calculated with the actual gene positions (blue curve) and with random gene positions (green curve). The points where the autocorrelation (blue curve) departs from the random signal (green curve) correspond to couples of genes, the change in expression levels of which are correlated (when the blue curve is above the green one) or anti-correlated (when the blue curve is below the green one).

The main characteristics of figures 2 and 5 are similar. Both bacteria share the steep decay of the averaged autocorrelation curve for inter-gene distances lower than 100 genes and two maxima at a distance of 200 and 650 genes. However there are some differences between *B.subtilis *and *E.coli *for long-range peaks since some of them are shifted: maxima at 1300 and 1500 in *B.subtilis *correspond to peaks at 1100 and 1400 in *E.coli*, respectively. The minimum at 900 in *B.subtilis *is shifted to 850 in *E.coli*. Some peaks and troughs, however, are specific to one specie such as those located at 1380, 1700 and 2180 in *E.coli *and at 550, 850, 1750–1900 and 2050 in *B.subtilis*. Probably due to the greater number of missing data the autocorrelation function is noisier for *E.coli *than for *B.subtilis*.

## Discussion

### Comparison of our results to already published observations

#### What has already been observed

The present study of gene expression data from *B.subtilis *and *E.coli *has allowed us to confirm and extend some previously published observations:

• We show for both bacteria that closely spaced genes exhibit highly correlated expression levels. This correlation decreases rapidly with oscillations having a period of 14.5 ± 1 genes corresponding to 14.5 ± 1 kb. Short-range correlations are obvious in the study by Sabatti et al [11] of gene expression data from *E.coli*. Jeong et al [9] have also observed short-range correlations up to 16 kb in their analysis of expression changes during replication in various *E. coli *strains.

• In this work the averaged autocorrelation function for *E. coli *may be seen as the resultant of two main oscillations (with periods of 557 ± 30 kb and 100 ± 18 kb). In *B. subtilis *we observe four oscillations (with periods of 600 ± 55 kb, 240 ± 21 kb, 113 ± 21 kb and 60 ± 6 kb). Rocha et al [17] analysed the distribution of the genes involved in sulphur metabolism in the genome of *E.coli *and found a number of them to be clustered into statistically significant islands located 650 kb apart. In their study of transcriptional activities in *E.coli*, Jeong et al [9] have observed significant correlations for genes located 690 kb or 523 kb apart (depending on physiological conditions) together with a clump of periods around 115 kb.

#### New results

• We show here that the long-range and short-range correlations are similar in *E. coli *and *B. subtilis*. That the observed regularities should be shared by two widely distant bacteria immediately suggests that it could be a property common to other bacteria as well.

• In addition, our results are indicative of an unexpected property that may well modify the current model of the nucleoid organisation: the changes in the level of expression of any gene are correlated (positively or negatively) to the changes in the expression level of other genes, located at well-defined long-range distances and regardless of their localisation on the chromosome in both organisms.

• The long-range periods of the autocorrelation function do not correspond to the 100 kb domain organisation, which may result from the control of topological constraints on the rotation of the double helix [12] and was observed in a study of the positions of genes that are controlled by a sequence-specific transcriptional regulator and the genes encoding this regulator [18]. They do not correspond either to the macro-domain of 1 or 2 Mb proposed by Niki et al [2] and by Valens et al [5]. As all the genes exhibit the same long-range correlations, the phenomenon cannot be explained by some process involving regulators. Conversely, the observations made by Jeong et al [9] may be the result of the general phenomenon observed in this study.

### Our interpretation

Gene transcription can occur only on the nucleoid surface. Thus the expression correlations that we observed imply that the involved pairs of genes lies on this surface. However all the genes cannot be on the nucleoid surface at the same time. Therefore depending on the external conditions and/or physiological requirements of the cell, different groups of co-expressed genes should be accessible to the transcriptional machinery. Such constraint seems hardly compatible with an unstructured spatial organisation of the chromosome. Similarly a disordered or random packing is very unlikely to result in the significant periodicities described above. Rather, our observations suggest that the nucleoid must be packed in a fairly structured way.

#### Knowledge about the nucleoid and ribosomes sizes

The genome sizes of *E. coli *and *B. subtilis *are respectively 4.6 Mb (4425 genes encoding proteins) and 4.2 Mb (4108 genes encoding proteins). Half of the genes belong to an operon. The operons have an average size of three genes [12,19]. The nucleoid (the chromosome) shows up as a cylinder of approximate size of 0.5 × 0.7 *μ*m [12,20]. Its circumference of 1.5 *μ*m corresponds approximately to 16 kb of uncoiled DNA, or 16 genes. The diameter of a ribosome is 0.025 *μ*m [21], hence 25 to 30 ribosomes can be juxtaposed along the cylinder length of 0.7 *μ*m.

#### The possible chromosome configuration

**Figure 6:**
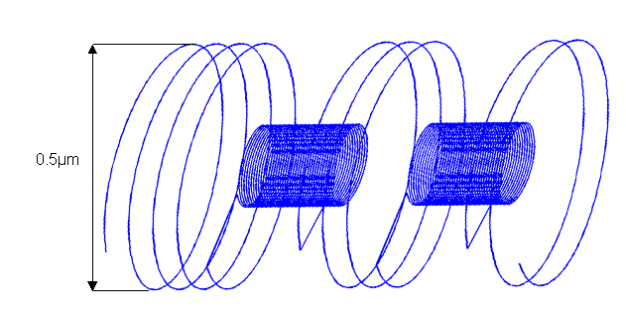
**The possible chromosome configuration. **We assume that the nucleoid structure consists of a solenoid with two types of spirals: • Large spirals of uncoiled DNA, containing the genes that are transcribed, that lie on the surface of the nucleoid and define its diameter (0.5 *μ*m). • Small spirals of coiled untranscribed DNA that lie inside the nucleoid.

We assume that the nucleoid structure consists of a solenoid with two types of spirals (figure 6):

• Large spirals of uncoiled DNA, containing the genes that are transcribed, that lie on the surface of the nucleoid and define its diameter.

• Small spirals of coiled untranscribed DNA that lie inside the nucleoid.

Cellular elements, in particular the ribosomes on the surface of the nucleoid, impose limits to the number of large expressed spirals. The distance between two large spirals cannot be shorter than the diameter of the ribosome; hence a maximum of 25 to 30 uncoiled DNA large spirals may stand on the nucleoid surface (see knowledge about the nucleoid and ribosomes sizes).

Short-range correlations show that contiguous co-expressed genes do not span more than 100 kb, hence no more than 6 large spirals. We can therefore assume that the average length of contiguous uncoiled DNA is 3 large spirals (see figure 6). This will make 8 to 10 groups of three consecutive large DNA spirals distributed along the chromosome.

#### Explanation of our results by this nucleoid representation

• The short-range correlations may be seen as resulting from two phenomena:

- The co-ordinated expression of the genes within operons. This explains the correlations in the expression of pairs of genes that are less than 5–6 genes apart.

- The presence of one or more consecutive DNA large spirals of approximately 16 genes on the nucleoid surface. The 14.5 ± 1genes period observed in the variations of the autocorrelation function points to those genes that belong to successive spirals and lie on a generatrix of the nucleoid cylinder.

• For long-range correlations we find 10 maxima in *E. coli *and 11 maxima in *B. subtilis*. These maxima probably result from groups of large DNA spirals on the nucleoid surface.

However, such a static representation of the nucleoid does explain neither the alternating pattern of maxima and minima nor their positions.

#### The dynamic of the nucleoid: a phenomenon, which is not fully explained

The dynamic of the nucleoid structure corresponds to the shift between small spirals of unexpressed coiled DNA to large spirals of expressed uncoiled DNA, and *vice-versa*. The large spirals are present only when there is effective transcription [22]. The transcription process can explain some of our observations:

- Long-range anticorrelations can result from coil-coiled DNA in small spirals next to large expressed spirals. It has been shown indeed that the opening of the double-stranded DNA during transcription leads to waves of compression of those regions of the chromosome that are close to the transcribed DNA [23]. It can therefore be speculated that the expression of the genes in large spirals leads to the super-coiling of the neighbouring small spirals, hence to the impossibility of opening its DNA and to its transcription.

The pattern of maxima is more difficult to explain since it does not correspond to multiples of a single inter-gene distance. In the case of *B. subtilis *for example, the maxima are at inter-gene distances that are multiples of 650 and multiples of 650 plus 200 (200; 650, 850; 1300, 1500). We speculate that this pattern is a consequence of the dynamic of the nucleoid structure but we currently have no explanation for it. Current work is in progress to try to explain the maxima and minima of the correlation function, which is reminiscent of a beat phenomenon between two stable waves that could be generated by the transcription process.

## Conclusion

The analysis of gene expression data compendium provided information on the nucleoid organisation in circular double stranded DNA bacteria. Our results confirm and complete other observations like those obtained by microscopy. Co-expression variations of neighbouring genes on the chromosome suggest that large DNA spires of 14 to 16 genes length stay on the nucleoid surface. This estimation of a large spire length corresponds to the estimation by microscopy of the nucleoid circumference. The contiguous DNA on the nucleoid surface does not exceed around 100 genes (which is equivalent to 100 kb). This segment is organised in several large spirals of 14 to 16 genes.

The long-range correlation pattern is more surprising: the changes in level of expression for any gene are correlated (positively or negatively) to the changes in expression level of genes, located at well-defined long-range distances independently of their location on the chromosome. This original observation is based on the analysis of several independent sets of gene expression data, which put together a great variety of physiological conditions. However the long-range correlations do not correspond to the domains identified so far in the nucleoid. We are currently exploring a model where the long-range correlations could result from a beat phenomenon between compression and decompression waves generated by the transcription process.

## Methods

### Data used and normalisation

The microarray data sets have been downloaded from the following websites.

#### Bacillus subtilis

- Helmann et al [24] at the Stanford microarray database 

- Yoshida et al[25,26], Ogura et al [27,28], Kobayashi et al [29], Asai et al [30], Doan et al [31], Molle et al [32], and Watanabe et al [33] at KEGG expression database 

- Jarmer et al [34] at the Center for Biological Sequence analysis site 

#### Escherichia coli

- Mori et al [35] at KEGG expression database 

- Newton et al [36] at 

The data were normalised (mean equal to 0 and variance equal to 1) according to the experimental conditions (figure 1 part 1). They were concatenated for each organism leading to a file of gene expression levels made of 262 experimental conditions for *B. subtilis *and 106 experimental conditions for *E. coli*.

### Estimation of the correlations and the regularities (figure 1)

The aim of this article is to observe how gene co-expressions vary as a function of the inter-gene distance.

1. For each organism the co-expression among each pair of genes is evaluated with a non-parametric correlation: the Kendall tau [15,16] (figure 1 part 2).

To define the Kendall tau *τ*, we start with the *N *data points *(xi, yi)*, the expression levels of the genes *x *and *y *in the experimental condition *i*, respectively. Considering all the *1/2N(N - 1) *pairs of data points *(xi, yi) (xj, yj)*, we call a pair "concordant" if the differences *(xi-xj) *and *(yi-yj) *have the same sign and "discordant" if the differences have opposite signs. If *(xi-xj) *is equal to zero, we call the pair an "extra *y *pair." If *(yi-yj) *is equal to zero, we call the pair an "extra *x *pair." If both *(xi-xj) *and *(yi-yj) *are equal to zero the pair is ignored. Kendall's tau *τ *is the following simple combination of these various counts:



2. For each gene, we evaluate its distances from those other genes, the expression levels of which vary simultaneously. The variations of co-expression according to inter-gene distance (figure 1 part 3) are evaluated with the linear autocorrelation [16] on the gene's Kendall tau vector.

The autocorrelation for an inter-gene distance of *j *is calculated as followed:



with *y *the Kendall tau vector of a gene and  the mean of *y*, *N *the number of genes

Note that the bacterial chromosome is circular, so there is no boundary problem. Note that the distance between two genes used in this article is the difference their ranks on the chromosome (approximately equivalent to the number of kb).

### Signal deconvolution and estimation of the periodicities

The variation of co-expression according to the inter-gene distance is a superimposition of several periodicities (from small to large scale). To identify these periodicities the averaged autocorrelation signal was deconvoluated with Peakfit 4.06 (Jandel Scientific, San Rafael, CA). The percentage of the autocorrelation that this representation explains is calculated as follow:



with *y *the autocorrelation vector and *x *the signal generated by the sum of the deconvolution periodicities and *N *the number of genes.

## Authors' contributions

ASC collected the data, performed the statistical analyses and drafted the manuscript. AG and BT participated in the statistical analysis. AH conceived the study, participated in its analysis and coordination. All authors participated to the elaboration of the model, read and approved the final manuscript.
